# Validating Discovered *Cis*-Acting Regulatory Genetic Variants: Application of an Allele Specific Expression Approach to HapMap Populations

**DOI:** 10.1371/journal.pone.0004105

**Published:** 2008-12-31

**Authors:** Susana Campino, Julian Forton, Srilakshmi Raj, Bert Mohr, Sarah Auburn, Andrew Fry, Valentina D. Mangano, Claire Vandiedonck, Anna Richardson, Kirk Rockett, Taane G. Clark, Dominic P. Kwiatkowski

**Affiliations:** 1 Wellcome Trust Centre for Human Genetics, University of Oxford, Oxford, United Kingdom; 2 Wellcome Trust Sanger Institute, Hinxton, Cambridge, United Kingdom; 3 University Department of Paediatrics, John Radcliffe Hospital, Oxford, United Kingdom; 4 JDRF/WT Diabetes and Inflammation Laboratory, Cambridge Institute for Medical Research, University of Cambridge, Addenbrooke's Hospital, Cambridge, United Kingdom; 5 Department of Public Health Sciences, Section of Parasitology, University of Rome La Sapienza, Rome, Italy; University College Dublin, Ireland

## Abstract

**Background:**

Localising regulatory variants that control gene expression is a challenge for genome research. Several studies have recently identified non-coding polymorphisms associated with inter-individual differences in gene expression. These approaches rely on the identification of signals of association against a background of variation due to other genetic and environmental factors. A complementary approach is to use an Allele-Specific Expression (ASE) assay, which is more robust to the effects of environmental variation and *trans*-acting genetic factors.

**Methodology/Principal Findings:**

Here we apply an ASE method which utilises heterozygosity within an individual to compare expression of the two alleles of a gene in a single cell. We used individuals from three HapMap population groups and analysed the allelic expression of genes with *cis*-regulatory regions previously identified using total gene expression studies. We were able to replicate the results in five of the six genes tested, and refined the *cis*- associated regions to a small number of variants. We also showed that by using multi-populations it is possible to refine the associated *cis*-effect DNA regions.

**Conclusions/Significance:**

We discuss the efficacy and drawbacks of both total gene expression and ASE approaches in the discovery of *cis*-acting variants. We show that the ASE approach has significant advantages as it is a cleaner representation of *cis*-acting effects. We also discuss the implication of using different populations to map *cis*-acting regions and the importance of finding regulatory variants which contribute to human phenotypic variation.

## Introduction

Understanding the cellular mechanisms that modulate gene expression is fundamental to defining the genetic contribution to human phenotypic variation and disease susceptibility. Identifying non-coding regions that alter gene expression by affecting transcriptional efficiency or by modulating mRNA splicing is difficult. *Cis*-regulatory elements reside on the same chromosome as the gene they regulate, and act only on the copy of the gene on the same chromosome. These elements are usually located close to the transcription starting site but can be located hundreds of kilobases (kb) from the gene they regulate[2], [3]. Trans-regulatory elements may be located on a different chromosome and regulate both copies of the gene. Absolute expression levels are a composite reflection of many genetic and environmental variables. Dissecting out the relative contribution of a single regulatory element for a gene influenced by multiple processes is challenging. A secondary challenge is to identify the causal genetic variant from other variants, in regions where linkage disequilibrium (LD) is high.

Numerous genome-wide scale investigations of total gene expression using genome-wide high density genotyping and classic linkage/ association mapping have contributed to the identification of putative *trans* and *cis*-acting regulatory variants [3]–[8]. These approaches offer the advantage of simultaneously analyzing thousands of genes by using gene expression arrays. However they are limited by the potential inter-individual environmental and genetic differences that may confound results[9].

An alternative method to the mapping of regulatory variants is to use an allele specific expression (ASE) approach that compares the relative expression of the two alleles in the same individual [10], [11]. This approach can be applied if the gene under study has polymorphisms in the transcript, such as single nucleotide polymorphisms (SNPs). These polymorphisms can be used to quantify the relative expression of the two alleles in heterozygous individuals; if the expression of the alleles is not equal (allelic imbalance), it suggests that the expression of the gene is under *cis*-regulation. As both alleles are exposed to the same environmental, technical and genetic factors (e.g. *trans*-acting effects), relative abundance of allele specific transcript will reflect *cis*-acting effects only.

Previously we have described the identification of a long range *cis*-regulator for *IL13* [2], located using ASE mapping methodology in CEU (Utah residents with ancestry from northern and western Europe) HapMap cell lines[12]. Here we apply a robust *cis*-mapping approach (described in the results) to validate highly significant *cis*-acting variants previously identified by Cheung *et al* [5] in whole genome linkage/association-based studies of total gene expression. We use the same panel of CEU lymphoblastoid cell lines (LCL) and we extend the analysis to other HapMap sample sets (Han Chinese from Beijing (CHB) and Yoruba from Ibadan (YRI)). We discuss the advantages and drawbacks of the two approaches and the implications of mapping regulatory variants in populations with greater genetic diversity.

## Results

We applied ASE to a set of genes (Table 1) with highly significant evidence for *cis*-regulation from total expression data [5], [7]. Gene selection was based on: (1) the presence of a transcribed SNP, as this is required to measure relative transcript abundance (2) modest/high minor allele frequencies (MAF) of transcribed SNPs, to maximise the number of heterozygous individuals available for ASE analysis 3) PCR-primer design that can be applied to both cDNA and DNA samples, so that relative abundance of genomic DNA can be ascertained and used as a control 4) PCR-Primers that do not include other SNPs to avoid differential primer hybridization that could confound results. We selected the six genes that passed all the criteria and had a high MAF at the transcribed SNP in at least one of the HapMap populations: *IRF5*, *LRAP*, *CHI3L2*, *HSD17B12*, *POMZP3* and *AKAP10*.

**Table 1:** Genes and transcribed Single Nucleotide Polymorphisms (SNP) used in ASE assay.

Chromosome	Gene	Coding SNP	Position (ENSEMBL)	Alleles	Heterozygous Frequency		
					CEU	CHB	YRI
17	*AKAP10*	rs2108978	19802050 (Non-Synonymous)	C/T	0.483	0.289	0.559
1	*CHI3L2* a	rs13721	111585505 (Non-Synonymous)	C/A/T	0.6	0.44	0.04
1	*CHI3L2* a	rs7542034	11585504 (Synonymous)	G/A	0.036	0.044	0.322
11	*HSD17B12*	rs1061810	43834510 (3′_UTR)	C/A	0.317	0.378	0.483
7	*IRF5* b	rs2070197	128376236 (3′_UTR)	C/T	0.38	0.0	0.06
5	*LRAP*	rs1056893	96271195 (Synonymous)	C/T	0.433	0.556	0.417
7	*POMZP3*	rs1056119	76093427 (5′_UTR)	C/T	0.4	0.27	0.55

a Different transcribed SNPs were used to obtain ASE data for the CEU/CHB and YRI datasets due to MAF differences in the populations used.

b Only the CEU population was analysed due to the low MAF observed for transcribed SNPs in the YRI and CHB populations.

An allelic expression dataset was generated from the HapMap panel of LCLs from CEU, YRI and CHB unrelated individuals (total of 150 individuals). We performed cell culturing, RNA extraction, cDNA synthesis and allele specific quantification as described in the Material and Methods. Data were generated from two independent cultures (biological duplicates). Each biological duplicate consisted of 9 technical replicates. The overall experimental variability was very low, with low variance between technical replicates (mean coefficient of variance of 6%) and a high correlation between biological duplicates (mean Pearson correlation coefficient r = 0.85). These results support the assumption that ASE patterns presented here represent a biological phenomenon, being little influenced by variations in cell culture and other experimental techniques. Allelic expression imbalance (AEI) was determined if independent replicate assays showed allelic expression ratios that deviated from the ratios observed for genomic DNA. Using the observed variability between technical and biological replicates, we established an average sensitivity limit of 1.2 for detecting allele specific imbalances. Any results below this allelic expression ratio limit could be the result of experimental noise.

**Figure 1:**
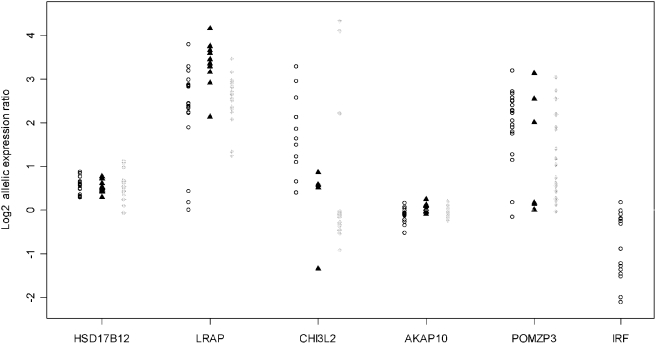
Allelic expression ratios (Log2) in CEU, CHB and YRI samples. Horizontal line represents a ratio of 1 (log2 = 0), where the expression of each allele of a gene is equal. Populations are represented by (о) CEU, (▴) CHB and (⧫) YRI. Number of samples tested: *HSD17B12* (CEU = 17, CHB = 12, YRI = 22 ), *LRAP* (CEU = 15, CHB = 11, YRI = 22), *CHI3L2* (CEU = 17, CHB = 6, YRI = 15), *AKAP10* (CEU = 21, CHB = 8, YRI = 27), *POMZP3* (CEU = 20, CHB = 5, YRI = 24), *IRF5* (CEU = 13). For the CHI3L2 we observed individuals with monomorphic expression (CEU = 6,YRI = 2) which are not represented in the graphic.

Of the 6 genes selected for this study (*IRF5*, *LRAP*, *CHI3L2*, *HSD17B12* and *POMZP3*, *AKAP10*), we observed AEI in the first 5 genes (Figure 1). For some genes, and in certain populations, allelic imbalances were detected in all the tested individuals. This was the case for the *LRAP* gene (in the YRI and CHB panels), the *HSD17B12* (in the CEU and CHB) and the *CHI3L2* (CEU and CHB). For the *AKAP10* gene we observed allelic expression ratios close to 1 in all populations, even in the CHB population where there is reported evidence for *cis*-regulation [7]. In the *CHI3L2* gene we identified some individuals that show detectable expression of only one allele (35% of the CEU and 13% of the YRI samples).

To map putative *cis*-acting variants associated with the observed AEI observed we correlated the ASE data with surrounding SNPs across approximately 200 kb 5′prime and 3′prime to the gene. If a candidate SNP has a *cis*-acting effect on the expression of the tested gene, AEI will only be observed in individuals heterozygous at that candidate SNP [2], [13]. The direction of the imbalance will depend on the phase relationship between the alleles at the candidate SNP and at the transcribed SNP. In particular, assuming an ASE ratio of allele 1 and allele 2 at the transcribed SNP then, individuals that contain the over-expressed allele at the *cis*-regulatory SNP in phase with allele 1 will have ratio in excess of one; similarly, individuals with the over-expressed allele in phase with allele 2 will have ratio less than one. For each gene, we used HapMap haplotype data for the cell lines considered.

**Figure 2:**
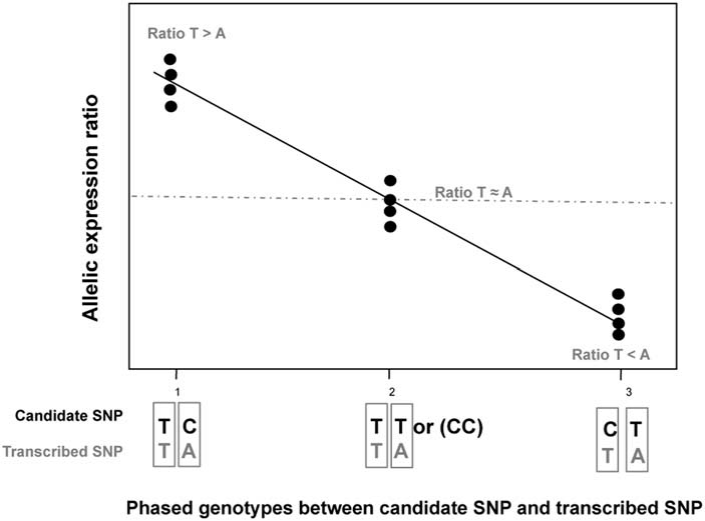
Statistical model for the detection of *cis*-acting variants. We applied a linear regression (LR) model ((similar to that of Teare *et al* [13]) to assess whether there was evidence of linear relationship between the allelic expression ratio of the phased heterozygous type 1 (TC/TA, group 1), combined homozygous types (TT/TA or CC/TA, group 2), and phased heterozygous type 2 (CT/TA, group 3). We assumed that homozygous types group will have no allelic imbalances.

For each candidate SNP we then used linear regression (LR) to assess whether there was evidence of an increasing or decreasing (linear) relationship between the ratio of expressions for the phased heterozygous type 1 (e.g. TC) (group 1), combined homozygous types (e.g. TT and CC) (group 2), and phased heterozygous type 2 (e.g. CT) (group 3) genotypes (see Figure 2). The statistical significance of the estimated slope between the group labels 1, 2, and 3 (x-axis) and allelic expression ratio (y-axis) is invariant to the labelling of the heterozygous groups. If the AEI data for a given transcript SNP is unidirectional showing overexpression of the same allele in all cell lines, the *cis*-acting variant is likely to be in high LD with the transcript SNP. If the AEI data is bidirectional, the *cis*-acting variant is likely to be in low LD with the transcript SNP. The three HapMap sample sets used (CEU, YRI and CHB) were analysed independently.

**Figure 3:**
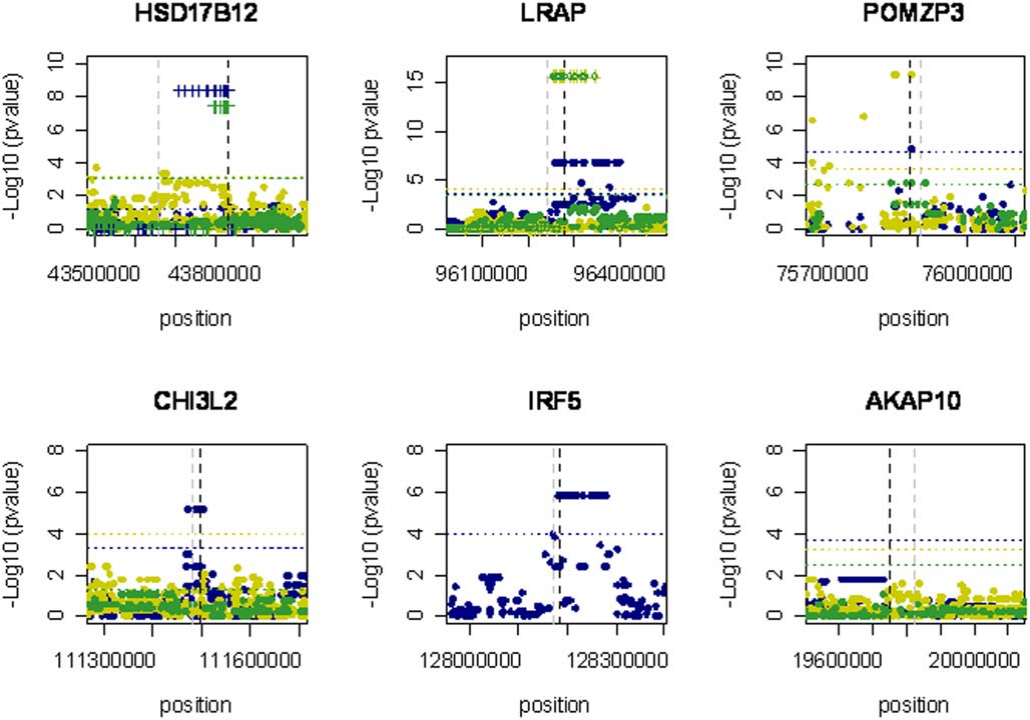
*Cis*-association of SNPs with ASE data. Hapmap populations used: CEU (blue), CHB (green) and YRI population (yellow) unrelated individuals. Dots represent the results using a LM and crosses represent SNPs associated with ASE data which are in complete LD with the transcribed SNP (T-test results). Coordinates are in NCBI Build 35. Horizontal lines reflect a multiple testing adjusted gene-wide statistical significance threshold of 5%. Vertical lines represent the 5′ prime (grey) and 3′ prime(black) of the gene.

In our data, we found *cis*-association in all of the 5 genes that showed differential allelic expression (Figure 3). Where *cis*-associations localised to a region of high LD, neighbouring SNPs showed similar levels of association.

For the genes where all tested individuals of a population demonstrated AEI in the same direction (see Figure 1), it is difficult to detect any regulatory variants using the LR model. However, we can assume that these individuals which have the same AEI phenotype will all be heterozygous at the putative regulatory SNP (will have the same haplotype) and test whether there is evidence of a deviation away from ratio one at those heterozygous SNPs. In this setting we use a one sample t-test, which is more robust to small sample sizes and minor deviations from the Gaussian distribution than the standard z-test. In addition we verify if imbalance results from the over-expression of the same allele at the regulatory SNP in all individuals, to eliminate false positives.

For *LRAP* and *HSD17B12* we observed overlapping *cis*-associations in all three populations (Figure 3). For *POMZP3* we found *cis*-association in two populations but for the CHB, there were insufficient samples for significant *cis*-mapping. In the *IRF5* gene we identified a large region with several *cis*-associated SNPs within the same haplotype block. These included the putative regulatory SNPs identified by Cheung *et al* [5], [14] and other SNPs found by others [3]. For the *CHI3L2* gene we found significant *cis*-associations in the CEU dataset, including the putative SNPs reported by Cheung *et al* [5], however no association using the same exonic SNP was found for the CHB data set, probably due to the small sample size. In the YRI samples while we still observed AEI, no significant *cis*-associations were detected for *CHI3L2*. All tested samples in this population were homozygous for the SNP found by Cheung *et al* [5], therefore eliminating the support to a functional role for this SNP in this population.

Combining all the unrelated individuals from the three populations in the analysis can increase the power to detect *cis*- effects on allele expression that are common, but of small magnitude. Stranger *et al* [3] recently showed that pooling total gene expression data from four Hapmap populations assisted in detecting smaller regulatory effects that are shared across populations. We performed the same analysis (LR model) using a combination of any 2 populations, as well as using the 3 populations. For the *CH3IL2* gene we did not use the YRI data, since a different transcribed SNP was used to determine the ASE results for the CEU and CHB datasets.

We cannot pool the samples without applying appropriate corrections, as population differentiation can produce spurious associations. We applied a stratified linear regression, and performed conditional permutations to control for the inflation of p-values, where data from an individual of a given population were assigned only to another individual of the same population [3].

**Figure 4:**
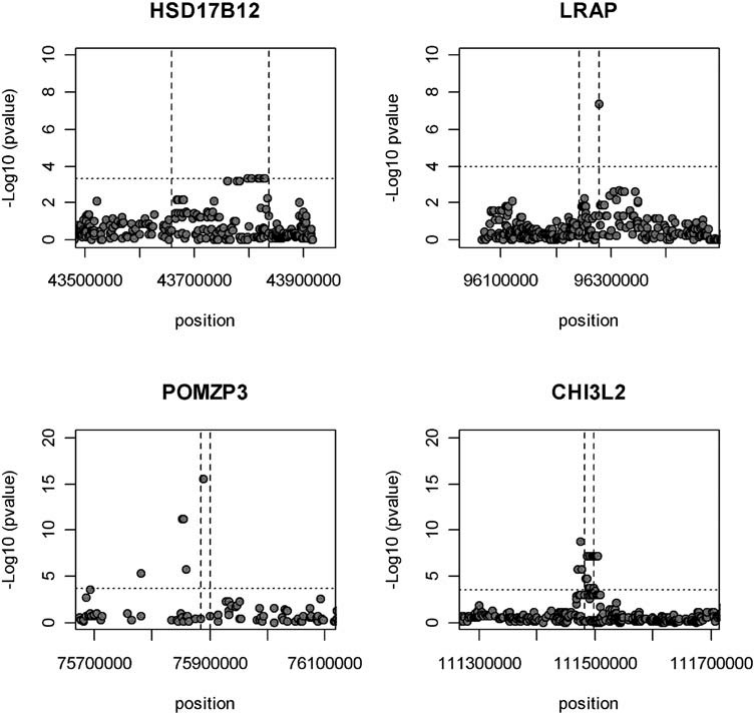
Multi-population *cis*-mapping (CEU, CHB, YRI). For the CHI3L2 gene, only CEU and CHB populations were pooled. Horizontal lines reflect a multiple testing adjusted gene-wide statistical significance threshold of 5%.

The results obtained by using multi-populations overlap with the single-population analysis (Figure 4). Due to the increase in the sample size, the associated *cis*-effect DNA regions when using the 3 populations were smaller than those detected by using single-populations.

## Discussion

Using the ASE approach we observed allelic expression imbalance for five of six candidate genes described by Cheung *et al* [5]. By mapping the allelic expression data we confirm the effect at the *cis*-acting polymorphisms identified by Cheung for these genes but these SNPs did not always carry the strongest effect. By using a more recent higher density SNP genotyping database we identified strong association with others SNPs in the vicinity of the polymorphism identified by Cheung *et al* [5], analogous with recently published data[3], [15].

As previously reported, repeating the analysis in other populations can allow the confirmation of an association signal. Using a population with low LD may help in localising the *cis*-acting signal to a smaller number of variants and therefore aid downstream analysis. Here we have verified that the association signals detected replicate very well across populations, even though the populations are divergent and the sample sizes are small. The only exception was for the *CHI3L2* gene, for which AEI was observed in both the YRI and CHB populations, but no statistically significant association was identified with neighbouring SNPs. By comparing the results from the 3 populations that have different LD structures particularly the YRI, where LD is lowest, we were able to localise the association to a smaller region for both *LRAP* and *HSD17B12* genes.

An important feature of the ASE approach is its potential to detect *cis*-acting effects, as this approach is robust to environmental confounders and trans-acting effects. It is possible to map *cis*-acting polymorphisms even using a low number of individuals using this method; eleven individuals in the CHB population were sufficient to accurately map the effect at the *LRAP* gene. Currently, cell lines with genotyping data are limited in number. ASE in addition can only be applied to individuals with a heterozygous transcript SNP which limits sample size. To increase statistical power others have pooled samples from different populations and we have repeated this approach using ASE.

Another limitation of the ASE approach relates to the need to use transcribed SNPs. Although others have attempted to use intronic SNPs, the results are less successful and genes have to be highly expressed in LCLs for effective results [15].

For *LRAP*, *CHI3L2* and *POMZP3* we could identity a main SNP effect using the three (two for *CHI3L2*) populations pooled together (rs2910686, rs2764546 and rs2005354 respectively). Refining the *cis*- associated regions can overcome the difficult task of isolating the causal regulatory variants that may be in LD with many others. Although combining different populations in a single analysis has advantages, there are implications we must consider as haplotype patterns between candidates SNP identified and the functional SNP itself may vary between populations and therefore confound results.

Evidence of *cis*-regulation has been reported for the *AKAP10* gene by Spielman *et al* [7] only for the CHB and JPT whereas no observation was found in the CEU data set. In addition Stranger *et al* [3] only found *cis*-association for this gene when pooling samples from different populations together. We did not observe AEI in the *AKAP10* gene using a total of 54 samples, and the SNPs reported previously to be associated clearly did not influence the allelic expression data in our study; in particular, individuals heterozygous or homozygous at the associated SNPs had similar allelic ratios (very close to one). It is possible that previous published expression data has been confounded by factors that not affect ASE for this gene.

Allele specific expression and total gene expression offer complementary approaches to identifying putative *cis*-regulatory mechanisms of gene expression. ASE when available on a high-throughput platform, similar to the method recently described by Serre *et al* [1], may have significant advantages over total gene expression data as it is a cleaner representation of *cis*-acting effects. Applying these assays to find regulatory variants which contribute to human phenotypic variation, such as responses to disease, can provide candidate regions for future investigation in fine-scale association studies of human disease.

## Materials and Methods

### Samples and RNA/cDNA preparation

Lymphoblastoid cell lines for 60 HapMap CEU, 60 HapMap YRI and 30 HapMap CHB were obtained from the Coriell Cell Repositories. Cell lines were cultured as described previously [16]. Total RNA was extracted with the RNeasy Mini-Kit (Qiagen) and treated during the process with RNase-free DNase I (Qiagen). Synthesis of First-Strand cDNA was processed according to the StrataScript™ First-Strand Synthesis System (Stratagene). For the experimental reaction we used 10 µg of total RNA.

### Allele specific transcript quantification

Informative exonic or untranslated SNPs for each gene were primarily selected from HapMap data (http://www.hapmap.org/). Where no informative SNPs were available at the HapMap data, additional transcribed SNPs were genotyped. Primers were designed using the dedicated software Spectrodesigner (Sequenom) and the same designs were applied to cDNA and genomic DNA samples. All primer designs are available upon request. The Allelotype platform from Massarray (Sequenom) was utilized for the accurate relative quantification of allele specific cDNA species. For each of the cDNA and genomic DNA assays, we performed 9 technical replicates. Biological replicates from independent cultures were assayed equally. For a given cDNA or gDNA assay the allelic transcript ratio was calculated on each of the 9 technical replicates from the relative quantity of the two allele-specific transcripts. The mean allelic transcript ratio for the whole assay was then calculated, and normalised to the mean allelic transcript ratio for the genomic controls.

### HAPMAP haplotypes

Haplotypes were downloaded from the HapMap Phase II database (http://www.hapmap.org). For data analysis we used all the Hapmap SNPs within 200 Kb of the transcribed SNP; monomorphic SNPs were excluded from the analysis. The minimum number of SNPs tested in each gene was: *IRF5* 750, *LRAP 686*, *CHI3L2 563*, *HSD17B12 406*, *POMZP3 660* and *AKAP10 572*.

Locations of the SNP markers were based on those of the human reference sequence (http://genome.ucsc.edu) of May 2004 (hg 17, build 35). Phase software was used to reconstruct haplotypes to include the SNPs not present in the HapMap data [17].

### Statistical analysis of allelic expression

For the association analysis, log2-transformed ratios of allele expression values for individuals with the heterozygous genotype at the transcribed SNP were used. To map putative *cis*-variants we used a statistical analysis approach based on the linear regression method (similar to that of Teare *et al* [13]). For genes where all individuals showed AEI, at SNPs where all samples are heterozygous, we applied a t-test to assess if there is a deviation from ratio 1. Additionally we confirm if AEI were cause by the over expression of the same allele among the samples tested for a given gene. For each gene, we performed 1000 permutations to determine a gene-wide significance p-value threshold [18] that corresponds to an overall false positive rate of 5%. All analyses were performed using the R statistical package. The ASE mapping R script is available upon request.
